# Evaluation of the RESIST-4 O.K.N.V immunochromatographic lateral flow assay for the rapid detection of OXA-48, KPC, NDM and VIM carbapenemases from cultured isolates

**DOI:** 10.1099/acmi.0.000031

**Published:** 2019-07-01

**Authors:** James Wesley MacDonald, Vindana Chibabhai

**Affiliations:** ^1^ Department of Clinical Microbiology and Infectious Diseases, University of the Witwatersrand, Johannesburg, South Africa; ^2^ Clinical Microbiology Laboratory, Charlotte Maxeke Johannesburg Academic Hospital, National Health Laboratory Service, South Africa

**Keywords:** carbapenemases, RESIST-4 O.K.N.V, OXA-48, KPC, NDM, VIM

## Abstract

**Purpose:**

This study aimed to evaluate the performance of the RESIST-4 O.K.N.V immunochromatographic lateral flow assay for the detection of OXA-48, KPC, NDM and VIM carbapenemases in 100 clinical *Enterobacteriaceae* isolates using solid culture media.

**Methodology:**

In total, 100 clinical *Enterobacteriaceae* isolates with characterized *β*-lactamase enzymes (OXA-48 *n*=46, KPC *n* =4, NDM *n* =43 and VIM *n* =10) were evaluated using the RESIST-4 O.K.N.V assay. The assay was also evaluated using carbapenem-sensitive control strains and confirmed non-carbapenemase-producing *Enterobacteriaceae* clinical isolates resistant to carbapenems. Inter-rater agreement of the test was evaluated by four different users who tested 11 randomly selected isolates daily over 3 days.

**Results:**

Overall accuracy of the assay was 99.5  %. For the detection of KPC, OXA-48 and its variants and VIM the assay correctly identified 100  % of the isolates when compared to PCR. Initial performance for NDM detection was sensitivity=95.3 %, specificity=100  %. Two PCR positive *Providencia rettgeri* isolates rendered false negative results on the assay. Retesting from a carbapenem zone of inhibition rendered a positive result for both isolates increasing the sensitivity to 100  %. No false positive results or cross reactions were detected.

**Conclusion:**

The RESIST-4 O.K.N.V is reliable, sensitive and specific for the detection of OXA-48, KPC, NDM and VIM carbapenemases. Further evaluation on improving NDM detection in organisms from the Proteeae tribe is warranted to determine optimal test conditions.

## Impact statement

This paper reports on the evaluation of the RESIST-4 O.K.N.V immunochromatographic lateral flow assay. This technology allows for the rapid detection of OXA-48 and its variants, KPC, NDM and VIM carbapenemases. This technology would allow clinical microbiology laboratories that do not have access to other molecular methods to rapidly detect these enzymes for guiding treatment as well as for infection prevention and control purposes.

## Introduction

*Enterobacteriaceae* are amongst the most common causes of hospital- and community-acquired bacterial infections in humans [1–4]. To treat these infections *β*-lactam antibiotics are often used. Carbapenems, a class of *β*-lactams, are broad spectrum agents used for the treatment of multi-drug-resistant *Enterobacteriaceae ,* including extended spectrum *β*-lactamases (ESBLs). The first description of enzymes capable of hydrolysing this class of antimicrobials (carbapenemases) were the chromosomally based genes in Gram-positive bacilli [5]. In the 1980s, the metallo-β-lactamases (MBL) were described in Gram-negative bacteria. The 1990s, however, saw the recognition and worldwide spread of plasmid-mediated carbapenemase enzymes in multiple species of organisms isolated from clinical samples [5].

### Classification of carbapanemases

Carbapenemases can be classified based on their molecular structure according to the Ambler classification system. Class A and D enzymes require serine at their active site while class B enzymes, the MBLs, require zinc ions in order to hydrolyse *β*-lactam antibiotics [5, 6].

*Klebsiella pneumoniae* carbapenemases (KPC); Guiana extended spectrum (GES); imipenem resistant (IMI); non-metallo-carbapenemases-A (NMC-A); *Serratia marcescens* enzyme (SME) and *Serratia fonticola* carbapenemase (SFC) are some of the notable examples found in class A [5, 6]. KPC is currently the most clinically significant group A enzyme. KPC-producing organisms are seen in North America and Europe with sporadic outbreaks in other parts of the world [7].

The class B MBLs consist of a complex group of enzymes that can hydrolyse all *β*-lactam antibiotics spare the monobactams and are not inhibited by *β*-lactamase inhibitors. The first description of a *bla*_NDM-1_ *K. pneumoniae* isolated in a Swedish patient who received healthcare in New Delhi, India was reported in 2008. Since that time there has been a global dissemination of NDM (New Delhi metallo-*β*-lactamase). In areas where the enzyme is endemic, such as India and Pakistan, it can predominate over other carbapenemases [5, 6]. Verona integron-encoded MBL (VIM) has been mainly identified in *Pseudomonas aeruginosa* but is also seen in the *Enterobacteriacae*. VIM-2 is the most commonly reported MBL worldwide with endemic spread in Greece, Spain, Italy and Southeast Asian countries such as South Korea and Taiwan. On the African continent, South Africa, Ivory Coast and Tunisia have also reported outbreaks of VIM-producing organisms [7].

Class D OXA *β*-lactamases are a heterogenous group of enzymes. The OXA-48 and its variants are increasingly being found in *Enterobacteriaceae* [5]. OXA-48 enzymes are capable of hydrolysing penicillins at a high level and carbapenems at low levels. It can spare the extended spectrum cephalosporins. However, in the presence of multiple ESBLs, organisms may become resistant to all *β*-lactams [5, 6, 8]. These characteristics result in difficult detection with standard phenotypic tests [8, 9]. First reported in Turkey in 2003 the OXA-48 producing isolates are now widely distributed throughout Europe, the Middle East and Africa [7].

Many countries report an assortment of carbapenemases circulating at any given time. An example of this is a South African laboratory-based surveillance report (GERMS-SA) for the period July 2015 to December 2017. During this period, 581 unique clinical isolates were submitted as part of surveillance testing. Carbapenemase genes (*bla*_NDM1/2_, *bla*_KPC_, *bla*_OXA-48_ and its variants, *bla*_GES_, *bla*_IMP_ and *bla*_VIM)_ were detected in 84 % of these isolates (487/581). *K. pneumoniae* was the predominant organism isolated (*n*=457; 79 %) followed by *Enterobacter cloacae* (*n*=49; 9 %), *S. marcescens* (*n*=30; 5 %) and *Escherichia coli* (*n*=19; 3 %). NDM (212/581; 36 %) and OXA-48 or variants (252/581; 43 %) were the two most prevalent carbapenemases detected, with VIM enzymes being reported less frequently [10].

### Rationale for routine identification of the carbapenemase gene

We are entering an era of individualized therapy based on the infection’s source, severity and information based on susceptibility studies of the bacteria [11]. For this reason, the rapid and accurate detection of carbapenemase-producing *Enterobacteriaceae* (CPE) in the routine diagnostic laboratory is essential not only for epidemiological and infection control reasons but also because it is necessary for the correct choice of antibiotic therapy [1, 2, 12]. However, routine identification of carbapenemases is not currently recommended by The Clinical and Laboratory Standards Institute [13].

Current treatment strategies of infections due to non-KPC-producing isolates have been largely based on case-control studies and cohort studies. In addition, patient outcomes differ depending on the implicated carbapenemase. The reported mortality in a matched case-control study associated with NDM infections is reported to be considerably lower (13 to 55 %) compared to that of KPC-associated infections (41 to 65 %). Numerous case reports of NDM-producing isolates also describe good clinical outcomes despite treatment with antimicrobials demonstrating *in vitro* resistance [14]. In general, however, the outcomes are worse for bacteraemic patients when infected with a carbapenemase-producing (CP) carbapenem-resistant *Enterobacteriaceae* (CRE) when compared to non-CP CRE [15].

Knowing which class of enzyme an organism produces could potentially guide therapy choices. Ceftazidime-avibactam is a combination of a third-generation cephalosporin and a new *β*-lactamase inhibitor (non-*β*-lactam). Avibactam inhibits class A (KPC and ESBLs) and class C enzymes as well as some of the OXA *β*-lactamases. However, it has no activity against the metallo-*β*-lactamases [11]. Meropenem-vaborbactam, is an agent that can be used against KPC producers but is not effective against MBL and OXA-48 producers [11]. Ertapenem can potentially be used as a ‘suicide drug’ in double carbapenem treatment combinations against KPC producers [11]. OXA-48 producers show high resistance towards temocillin, which would prevent its clinical use in such infections [11]. Aztreonam may potentially be used against MBL-producing isolates , provided they do not coproduce ESBLs [11].

With the emergence and rapid spread of CREs a considerable threat to clinical patient management and public health has evolved [1–4, 16]. In order to manage this threat, substantial efforts have been devoted to the development of an optimal diagnostic assay for the rapid detection of CREs. The characteristics of such an assay can be summarized by the ‘ASSURRED’ criteria described by the World Health Organization (WHO) for point-of-care tests. Such tests should be affordable, have a high sensitivity and specificity, be user friendly, deliver rapid results, have a long shelf life and not require sophisticated equipment, reagents or special disposal arrangements [17].

A solution to the need for rapid diagnostic assays for detection of CPEs, although not a point-of-care test, exists in the form of immunochromatographic lateral flow assays. Several variations on the lateral flow assays developed by Coris BioConcepts are commercially available. They can detect either single (OXA-48 or KPC) or multiple enzymes (OXA-48/KPC, OXA-48/KPC/NDM, OXA-48/KPC/NDM/VIM). Previous evaluations exhibited good performance of the assays with sensitivity varying between 94.4 and 100 % and specificity of 100 % when compared to molecular techniques for the single enzyme and triplex kit (OXA-48/KPC/NDM) [2, 9, 12, 16, 18–22]. Four other studies have evaluated the multiplex (OXA-48/KPC/NDM/VIM) assay, marketed as the RESIST-4 O.K.N.V, from cultured isolates [22–25]. These studies report a 100 % detection rate for KPC and OXA-48, 99–100 % for the detection of VIM and 83.3–100 % for the detection of NDM.

In this study, we describe the findings of an evaluation of the performance of the RESIST-4 O.K.N.V for detection of CPEs from isolates cultured on solid media.

## Methods

### Strain collection

Altogether, 100 clinical *Enterobacteriaceae* isolates with characterized *β*-lactamase content from the National Health Laboratory Services (NHLS) Infection Control Services Laboratory’s Isolate Bank (Charlotte Maxeke Johannesburg Academic Hospital, Johannesburg, South Africa) and the NICD’s Centre for Healthcare-associated Infections, Antimicrobial Resistance and Mycoses Unit’s Antimicrobial Resistance Laboratory and Culture Collection (AMRL-CC) (Sandringham, Johannesburg, South Africa) were selected for testing. *β*-lactamase genotypes were determined by multiplex PCR (LightCycler 480 II, Roche Applied Science, Germany) using the LightCycler 480 Probes Master kit (Roche Diagnostics, USA) and individual LightMix Modular kits (Roche Diagnostics, USA) by the NICD’s AMRL-CC. Carbapenemase genes detected by the PCR are *bla*_NDM1/2_, *bla*_KPC_, *bla*_OXA-48_ and its variants, *bla*_GES_, *bla*_IMP_ and *bla*_VIM_. The methodology of the PCR assay is found in a report by Perovic *et al*. [26].

**Fig. 1.**
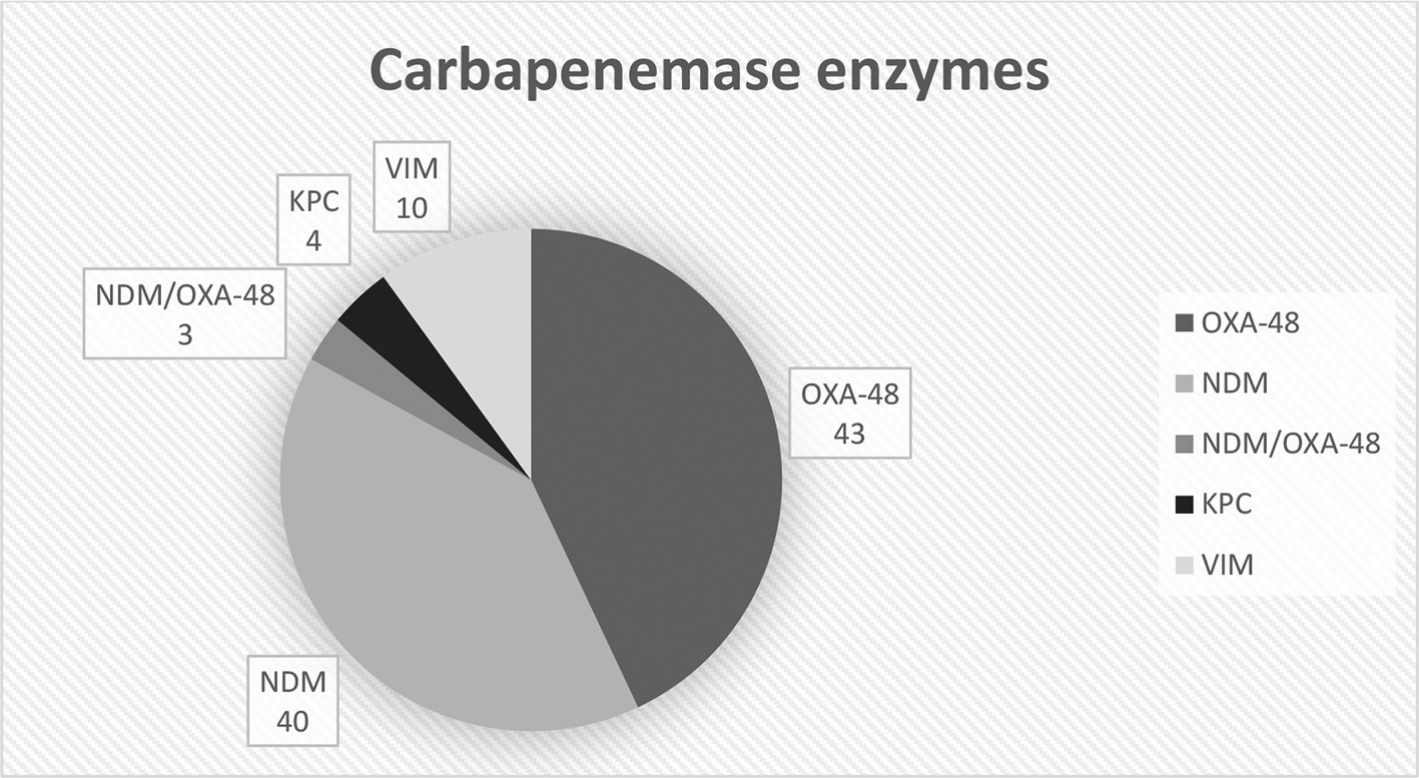
Total numbers for each carbapenemase enzyme tested. KPC=* K. pneumoniae* carbapenemases, NDM=New Delhi metallo-*β*-lactamase, VIM=Verona integron-encoded metallo-*β*-lactamase.

The breakdown of the carbapenemases evaluated is summarized in Fig. 1.

*K. pneumoniae* made up the majority of isolates (*n*=81). The remaining isolates consisted of *E. cloacae* (*n*=8), *Providencia rettgeri* (*n*=2), *Citrobacter freundii* (*n*=3), *E*. *coli* (*n*=3) and *S. marcescens* (*n*=3).

Isolates were plated out on Trypticase soy agar supplemented with 5 % sheep blood (5 % Sheep Blood agar) and MacConkey agar (Media Mage, Johannesburg, South Africa). Culture plates were incubated aerobically at 35 °C for 16–24 h. Testing was done from the 5 % Sheep Blood agar while the MacConkey agar was used to visually ensure that the isolates were not contaminated or mixed.

### RESIST-4 O.K.N.V

The RESIST-4 test was performed from colonies taken from the 5 % Sheep Blood agar after excluding contamination on MacConkey agar. Initial testing was performed as per the manufacturer’s instructions [27]. Briefly, one bacterial colony was taken from the 5 % Sheep Blood agar and homogenized in the LY-A buffer. Three drops were then added to the sample well of each of the two cassettes (KPC/OXA-48 and NDM/VIM). Results were recorded after 15 min.

### Precision of the kit

To evaluate the inter-rater agreement and reproducibility, four different users were asked to test 11 randomly selected isolates daily over 3 days. The users were blinded to the PCR results for all isolates. Isolates were refrigerated in between testing and testing was done from the same original culture plate. Users were supplied with the package insert for directions on how to use the test kit. Testing was done from the 5 % Sheep Blood agar. The samples that were selected at random consisted of NDM (*n*=6), OXA-48 (*n*=3) and VIM (*n*=2).

### Specificity

To confirm that the assay did not cross react with other *β*-lactamases/mechanisms of resistance, four clinical non-CPE isolates that were resistant to carbapenems were tested. Isolates were confirmed to be non-CPE by PCR (see above for specifications). In addition, the assay was tested using three carbapenem susceptible ATCC control strains. (* E. coli* ATCC 25922, *Enterobacter aerogenes* ATCC 13048, *K. pneumoniae* ATCC 1706).

### Statistical analysis

Sensitivity, specificity, positive predictive value (PPV) and negative predictive value (NPV) were calculated from all tested strains for each enzyme detected. Data was captured in 2×2 tables and analysed using Microsoft Excel 2016 (Microsoft). The following definitions for each parameter was used as defined by Leeflang and Allerberger [28].

- Sensitivity: the ability of the assay to identify the presence of the target carbapenemase. It is expressed as the percentage of isolates that did possess the target carbapenemase and tested positive [28].
- Specificity: the ability of the assay to recognize the absence of the target carbapenemase within an isolate. It is expressed as a percentage of the isolates that did not produce the target carbapenemase and tested negative [28].
- PPV: the ability of the assay to separate true positive results from false positive results. It is expressed as the percentage of isolates that tested positive for the target carbapenemase that indeed did express it [28].
- NPV: the ability of the assay to separate true negative results from false negative results. It is expressed as the percentage of isolates that tested negative for the target carbapenemase that truly did not express it [28].

## Results

### Performance

Table 1 displays the performance of the kit for each enzyme detected.

**Table 1.** Summary of the performance parameters of each enzyme tested

	OXA-48 *n*=46	KPC *n*=4	NDM *n*=43	VIM *n*=10		
**PPV** =TP/(TP+FP)	100,0	100,0	100,0	100,0		
**NPV** =TN/(FN+TN)	100,0	100,0	96,6	100,0		
**Sensitivity** =TP/(TP+FN)	100,0	100,0	95,3	100,0		
**Specificity** =TN/(TN+FP)	100,0	100,0	100,0	100,0		
**Accuracy** =(TP+TN)/(TP+FP+FN+TN)	100,0	100,0	98,0	100,0	**Overall accuracy**	99,5

All values are expressed as a percentage. The formulas used to calculate each test parameter are shown. Formal definitions for each parameter are described in Methods.

FN, false negatives (the number of isolates that tested negative for the target carbapenemase on the assay but were positive on PCR); FP, false positives (the number of isolates that tested positive for the target carbapenemase on the assay but were negative on PCR); KPC, *Klebsiella pneumoniae* carbapenemases; NDM, New Delhi metallo-*β*-lactamase; NPV, negative predictive value; PPV, positive predicative value; TN, true negatives (the number of isolates that tested negative for the target carbapenemase on both the assay and PCR); TP, true positives (the number of isolates that tested positive for the target carbapenemase on both the assay and PCR); VIM, verona integron-encoded metallo-*β*-lactamase.

### OXA-48

In total, 46 isolates tested positive for *bla*_OXA-48_ and variants by PCR. True positives=46, false negatives=0, false positives=0, true negatives=54.

### KPC

In total, four isolates tested positive for *bla*_KPC_ by PCR. True positives=4, false negatives=0, false positives=0, true negatives=96.

### NDM

In total, 43 isolates tested positive for *bla*_NDM1/2_ by PCR. True positives=41, false negatives=2, false positives=0, true negatives=57. The two false negative results belonged to two *P. rettgeri* isolates. These isolates were sent to the NICD’s reference laboratory for confirmatory testing and were confirmed to be NDM positive. The two isolates were plated out on 5 % Sheep Blood agar again and incubated as stipulated above. Repeat testing with a higher inoculum (five colonies) also yielded negative results. A 0.5 McFarland standard solution of the organisms was then used to streak a lawn of growth on 5 % Sheep Blood agar. Ertapenem (10 µg) and meropenem (10 µg) antibiotic disks were then placed on the plates and incubated for 24 h aerobically at 35 °C. Testing done from growth surrounding the zone of inhibition from both of the carbapenem disks using a 1 µl loop yielded positive results for NDM detection. This modification of the testing methodology increased the sensitivity and NPV for NDM detection to 100 %.

### VIM

In total, ten isolates tested positive for *bla*_VIM_ by PCR. True positives=10, false negatives=0, false positives=0, true negatives=90.

The assay consistently correctly identified the carbapenemase produced during the precision testing component of the study. Test results were not influenced by the age of the culture or the storage conditions of the agar (tested over 3 days from the same agar plate and refrigerated in between testing). The assay was reported to be user friendly by the different users.

No false positive results were obtained when control strains and confirmed CPE negative clinical isolates were tested.

## Discussion

We live in an age where antimicrobial resistance poses a real and ever-present threat to public health. With limited available antibiotics and few novel antimicrobials in development clinical microbiologists and laboratories are becoming more dependent on the rapid and accurate detection of resistance mechanisms.

In this study, the performance of this kit for KPC, VIM and OXA-48 and variants are in keeping with the other studies done on the RESIST-4 O.K.N.V and its predecessors. Other studies have also reported some detection problems with NDM [18, 22–24]. This study had three isolates that expressed both OXA-48 and NDM. The assay correctly identified both enzymes in all three isolates. Another study however did report false negative results for NDM in two of the three dual carbapenemase (OXA-48/NDM) producing isolates tested [24]. The excellent performance seen on detecting OXA-48 carbapenemases is a significant finding as it bridges the gap left by conventional phenotypic detection methods for these enzymes.

This report reiterates the description of false negative NDM results using either the RESIST-3 O.K.N or RESIST-4 O.K.N.V immunochromatographic lateral flow assays in organisms from the Proteeae tribe.

The study by Saleh *et al.* also reported false negative results for NDM in a *Proteus mirabilis* isolate [18]. This isolate repeatedly tested negative on the RESIST-3 O.K.N when sampled in areas of the plate away from the carbapenem disks. However, when the sample was taken next to a disk on Mueller–Hinton agar (MHA) or tested from tryptic soy agar with sheep blood the test was positive. Based on this finding the authors recommended an inoculum of 1 µl harvested adjacent to an ertapenem or meropenem disk be used for testing. The zinc content in the test agar was also believed to influence the detection capability of the test for NDM [18]. This same *P. mirabilis* isolate was also tested with the RESIST-4 O.K.N.V in a subsequent study done by Greissl *et al.* [23]. It again yielded a false negative result using standard testing methodology. Kolenda *et al.* evaluated the RESIST-4 O.K.N.V [22] and also reported a similar problem with the detection of NDM in two organisms (one *P. mirabilis* and one *Providencia stuartii *). These isolates were tested under three different conditions: firstly, a higher inoculum was used (five colonies versus one colony), secondly one colony grown on a selective medium (ChromID CARBA, bioMérieux) was used for testing, and lastly one colony harvested from around an ertapenem disk was used. However, only one of the isolates (* P. stuartii )* tested positive from the CARBA plate after 15 min.

The only two *P. rettgeri* isolates in our study cohort demonstrated false negative results. Carbapenem MICs as determined with diffusion gradient testing (Etest, Biomérieux) for both isolates were low (meropenem 0.12 mg l^−1^ and 0.25 mg l^−1^, respectively, and ertapenem <0.03 mg l^−1^ and 0.12 mg l^−1^, respectively). This may represent low levels of NDM expression in these strains. Saleh *et al.* suggested the same explanation for this observation made in their study. If so, the addition of the carbapenem disks would theoretically supply a sufficient environmental stimulus to induce carbapenemase production.

It is interesting to note that *P. rettgeri , P. stuartii* and *P. mirabilis* all belong to the same tribe of *Enterobacteriaceae ,* as proposed by Ewing (i.e. Proteeae) [29, 30]. Although only small numbers of Proteeae have been tested in this and previous evaluations, the kit performed suboptimally for this group of organisms without modification of test methodology. Further evaluation of the RESIST kits is required using higher sample numbers of Proteeae in order to optimize detection of NDMs.

**Fig. 2.**
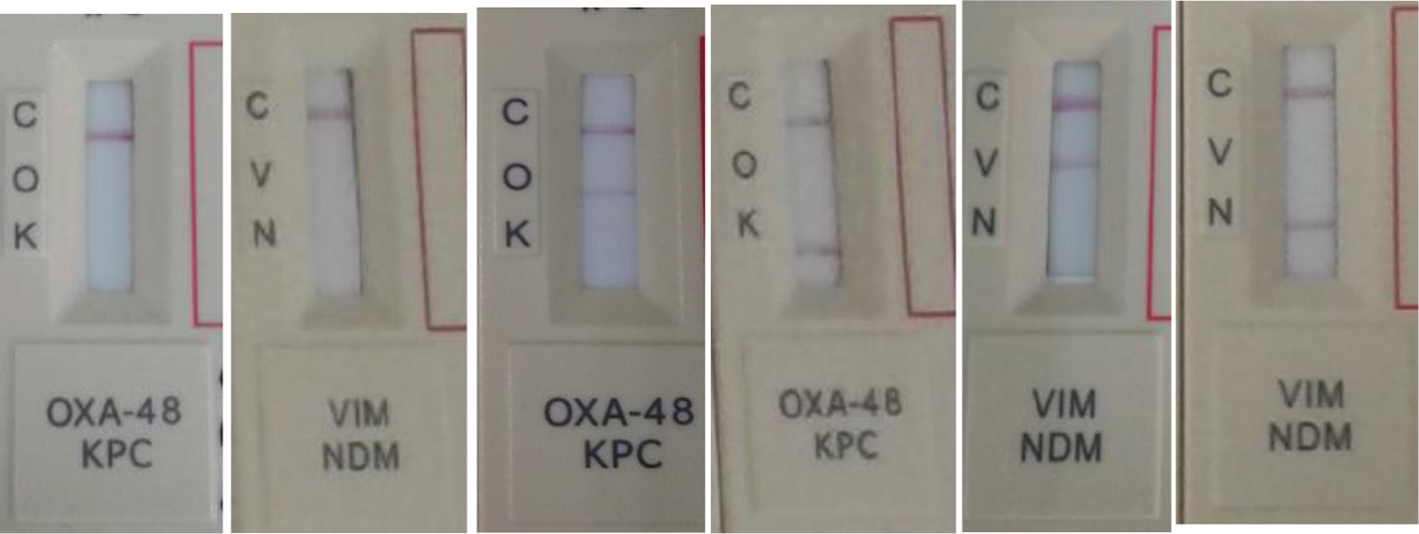
Expected visual results with assay. From left to right: negative for both OXA-48 and KPC; negative for both VIM and NDM; positive for OXA-48; positive for KPC; positive for KPC; positive for VIM; positive for NDM. C=control band; O=OXA-48; K=KPC; V=VIM; N=NDM.

In this assessment, the assay had an overall accuracy of 99.5 %. The assay was found to be easy to use and gave clear, easy to read lines for both cassettes (OXA-48/KPC and NDM/VIM) when tested from 5 % Sheep Blood agar. The assay was robust, and no false positives or cross reactions were detected. Testing from older refrigerated samples did not seem to affect the performance of the assay. The manufacturers do, however, recommend using fresh bacterial colonies for optimal test performance. The package insert indicates that a maximum time of 15 min be given to read results, but it was found that clear positive lines appear well before this time. Fig. 2 illustrates the expected results for the assay.

Limitations of this study include the relatively small number of organisms that were tested. The selection of organism genotype was chosen to simulate the epidemiology of CPOs currently seen in South Africa. The resistance genes carried by the isolates were not sequenced, which could potentially have given a better picture on other resistance mechanisms. It is however unlikely that such other mechanisms altered the results of this evaluation. The fact that the majority of isolates consisted of *K. pneumoniae *, and that we could not establish the genetic relationship between these isolates, could also possibly have skewed results. This is however also a reflection on the current epidemiology seen in South Africa, and elsewhere in the world. All the assays received for this evaluation belonged to the same LOT. We could thus not formally evaluate the assay for any possible batch-to-batch variation.

Future evaluations should aim at validating the performance of the assay on other routinely used agar media and other sample types used to screen for carbapenemase detection. Further species-specific performance evaluations of the assay are required to determine if the findings of this and other studies can be generalized to all NDM Proteeae isolates and to expand on the potential benefit of routinely adding antimicrobial disks/substances to testing conditions for improved detection. An additional study is currently being conducted in our setting to validate the assay’s performance on detection directly from urine samples.

The ease of use, reliable results with a high sensitivity and specificity and rapid turnaround time comply with the WHO ‘ASSURED’ criteria. This technology is thus an appealing addition to any clinical microbiology laboratory in both high-income and low-resource settings. Its use has the potential to significantly impact on management of CPE infections in the near future.
